# Adaptogenic Properties of a Phytoecdysteroid-Rich Extract from the Leaves of *Spinacia oleracea* L.

**DOI:** 10.3390/plants10122555

**Published:** 2021-11-23

**Authors:** Yuliya S. Sidorova, Vladimir A. Shipelin, Nikita A. Petrov, Sergey N. Zorin, Vladimir K. Mazo

**Affiliations:** Federal Research Centre of Nutrition and Biotechnology, 109240 Moscow, Russia; sidorovaulia28@mail.ru (Y.S.S.); petrov-nikita-y@mail.ru (N.A.P.); zorin@ion.ru (S.N.Z.); mazo@ion.ru (V.K.M.)

**Keywords:** stress, polyphenols, Wistar rats, ICR mice, exercise test, immobilization, memory, anxiety, catecholamines, acute toxicity

## Abstract

Increasing the ability of the human body to adapt in conditions of physical or emotional stress is promising from the standpoint of the use of preventive nutrition containing functional food ingredients (FFI) with proven effectiveness in complex physiological in vivo studies. In this work, we developed FFI from spinach leaves (*Spinacia oleracea* L.) with a high content of polyphenols and adaptogens—phytoecdysteroids. Using in vivo models of increased physical activity and immobilization-induced emotional stress, we evaluated the nonspecific resistance of rats in response to the addition of the developed FFI to the diet. In the acute toxicity experiment, we found no signs of FFI toxicity up to 5000 mg/kg body weight. As a result of the daily 26-day consumption of FFI, we observed an anxiolytic effect in physiological studies. FFI prevented an increase in the content of biogenic amines in the blood, the main markers of the stress system, and had a positive effect on the lipid metabolism of the rats. The obtained results demonstrate a “smoothing” effect on the body’s reaction in response to induced stress conditions.

## 1. Introduction

Nowadays, the daily impact of psychoemotional stress, the development of chronic fatigue syndrome, and increased physical and mental stress makes it necessary to use specialized food products (SFP) with proven adaptogenic effects in human nutrition [1]. The functional food ingredients (FFI) of SFP obtained from traditional food plants are able to form nonspecific resistance in the human body while avoiding the typical disadvantages of pharmacological drugs [2].

Phytoecdysteroids of medicinal plants such as *Panax ginseng, Eleutherococcus Senticosus, Rhaponticum Carthamoides, Rhodiola Rosea,* and *Schisandra Chinensis* are widely known as adaptogens [3,4]. Phytoecdysteroids are known for the numerous and diverse biological activities associated with their anabolic, adaptogenic, antidiabetic, hypolipidemic, and hepatoprotective effects [5,6]. Thus far, these results have positioned phytoecdysteroids as effective agents against several acute and chronic pathological conditions [7]. Of particular interest in this direction are poorly studied aspects of the synergism of phytoecdysteroid pharmacological effects and the metabolic action of minor biologically active substances. Such combinations are part of the composition of the traditional widely-used food plants in human nutrition, among which one of the popular representatives is spinach (*Spinacia oleracea* L.) [8,9]. The global production of spinach is over 25 million tons per year, making it an easily accessible and promising raw material for these purposes [10]. Spinach, traditionally known as a food product rich in vitamins and minerals [8], has been studied for decades for its neuroprotective [11,12], antihyperlipidemic [13], antiglycation [14], appetite suppressive [15], anti-osteoarthritic and chondroprotective [16], hepatoprotective [17], antioxidant [18], antiproliferative and anti-inflammatory properties [19]. Only few studies are devoted to the anxiolytic activity of spinach [20,21,22].

The presence in spinach of a unique profile of flavonoids and phytoecdysteroids explains the wide range of its biological activity. The chemical composition of spinach includes carotenoids, the phenolic compounds quercetin and kaempferol, derivatives of patuletin, spinacetin, spinatoside, jaseidin and flavone, as well as various phenolic acids, primarily ferulic and p-coumaric acid [23]. The content of 20-hydroxyecdysone in spinach leaves, is about 0.01%, which is an order of magnitude lower than in medicinal plants [3]. However, there are methods for concentrating phytoecdysteroids and extracting biologically active compounds (BAS), such as solid–liquid extraction, liquid extraction under pressure, and supercritical liquid extraction. The most effective method of extracting antioxidant and anti-inflammatory compounds from spinach leaves is liquid extraction under pressure [24]. Together with the use of technologies of concentration and ultrafiltration of low-molecular fractions, an increase of BAS in the FFI composition of more than 30 times compared to the raw material can be realized [25].

The use of in vivo models [26,27] in laboratory rodents makes it possible to reproduce various stress scenarios, such as hypothermia [28], increased physical activity [29], and emotional stress [30]. Laboratory animals, unlike humans, are characterized by instinctive behavior under a particular model, which is an advantage in preclinical testing on adaptogenic properties and permits the exclusion of many factors related to the heterogeneity of stress conditions in humans.

The present study aimed to evaluate the non-specific resistance of rats in light of the manifestation of pharmacological effects caused by the combined effect of phytoecdysteroids and other minor BAS included in FFI developed from spinach, under conditions of increased physical energy expenditure and immobilization-induced emotional stress.

## 2. Results

### 2.1. FFI from Spinacia oleracea L. Leaves: Polyphenol and Flavonoid Profile

By applying concentration technologies of and the ultrafiltration of low molecular weight fractions, it was possible to achieve a high concentration of polyphenols in the FFI composition. The total polyphenol content, determined spectrophotometrically using the Folin–Ciocalteu method, was 41.9 ± 4.1 mg-eq. of gallic acid/g. Phytoecdysteroid content (20-hydroxyecdysone, 20E) determined by HPLC-MS was 14.7 ± 1.5 mg/g. The flavonoid profile determined by HPLC-DAD is presented in Table 1.

### 2.2. Acute Oral Toxicity of FFI

Following a single oral administration of FFI at a dose of 5000 mg/kg of body weight, we did not observe the death of any mice, either male or female. In the first six hours after the administration of FFI, and subsequently for 13 days, all animals had normal appearance, stool, and appetite, were mobile, and gained body weight equally. Necropsy examination on the 14th day did not reveal any specific pathological changes in the internal organs. When assessing integral indicators (body weight dynamics, liver mass) as well as liver lipid profiles and corticosterone and prostaglandin E2 levels in the blood plasma, no negative changes were detected compared with the control animals (Supplementary Figure S1).

Thus, it was not possible to quantify the average lethal dose (LD_50_) of FFI for mice in this experiment; in any case, its value exceeds 5000 mg/kg of body weight, which makes it possible to classify FFI as a low-hazard substance (hazard class V) [31].

### 2.3. Experiment No. 1: Adaptogenic Properties of FFI in a Model of Immobilization-Induced Emotional Stress

#### 2.3.1. Integral Indicators

The results of the rats’ division into groups according to the “Elevated plus maze” (EPM) test along with the body weight of the animals are presented in Table 2.

**Figure 1:**
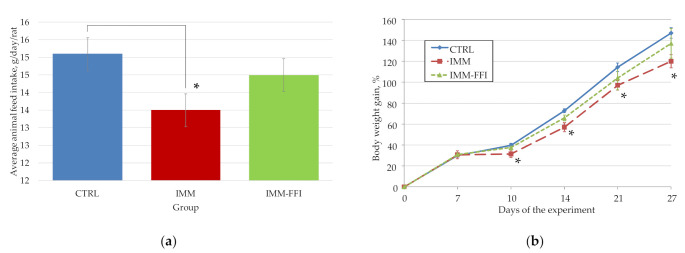
Integral indicators of rats during experiment No.1: (**a**) average animal feed intake, g/day/rat; (**b**) rats weight gain, %. *—Difference with the control group is significant, *p* < 0.05.

The general condition of all animals was satisfactory in terms of appearance, quality of wool, and food and water consumption during daily behavior checks throughout the experiment. The average daily feed consumption by the animals of the control (CTRL), IMM, and experimental (IMM-FFI) groups during the whole experiment is presented in Figure 1a. As shown, animals in the IMM group subjected to daily immobilization consumed significantly less feed compared to animals in the CTRL group (*p* < 0.05, Mann–Whitney test). The average feed consumption by animals of the IMM-FFI experimental group treated with FFI did not differ significantly from the indices of both control groups. The consumption of FFI by animals in the experimental group IMM-FFI was 84.0 ± 6.8 mg FFI per kg body weight/day, which corresponds to 1.4 ± 0.1 mg 20E per kg body weight/day and 199.4 ± 11.5 mg of total polyphenols in gallic acid equivalent/kg body weight. Figure 1b shows the dependence of the body weight of the animals in all groups for the duration of feeding. Already on the 10th day of the experiment, a significant decline in growth in the IMM group relative to the CTRL group was revealed (*p* < 0.05, Mann–Whitney test). The lag in the growth of animals in control group IMM is shown throughout the experiment, and may be due to both lower feed consumption and daily stressing immobilization. Significant differences in the body weight of the animals in the IMM-FFI group relative to both control groups were not detected. FFI consumption reduced the negative effects of immobilization stress to a certain extent.

#### 2.3.2. Memory Function and Behavioral Responses

**Figure 2:**
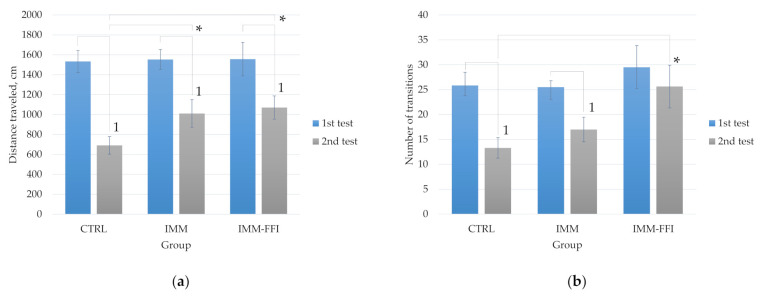

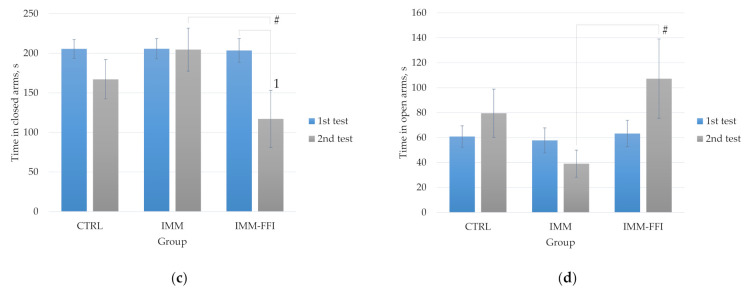
EPM testing results: (**a**) distance traveled in the maze, cm; (**b**) a number of transitions between maze arms; (**c**) time in closed arms, s; (**d**) time in open arms, s. First test before feeding by experimental rations. Second test on the 25th day of the experiment. **1**—Differences are significant (*p* < 0.05) compared to 1st test. *—Differences are significant compared to the CTRL group, *p* < 0.05. **#**—Differences are significant (*p* < 0.05) compared to the IMM group, *p* < 0.05.

Figure 2 shows comparative results of the EPM testing of animals at the beginning of the experiment and after 24 days of feeding with experimental diets. At the 25th day of the experiment, the behavior of the animals changed: the indicator of the distance traveled differed significantly for all groups compared to the first test (*p* < 0.05, Mann–Whitney test). Animals of the IMM and IMM-FFI groups exposed to daily immobilization moved significantly more through the maze during the second test. This is expressed in a significantly greater (*p* < 0.05, Mann–Whitney test) distance traveled compared to the animals of the control group (Figure 2a). In the IMM-FFI group, animals performed significantly (*p* < 0.05, Mann–Whitney test) more transitions between arms compared to the control group (Figure 2b). Greater locomotor activity of animals was apparently associated with daily immobilization. The animals of the IMM-FFI experimental group spent significantly less time in the closed arms of the maze compared to the first test (*p* < 0.05, Mann–Whitney test). They also spent significantly less time in closed arms (Figure 2c) in the second test and, accordingly, spent more time in open arms compared to the animals of the IMM control group (Figure 2d).

Short-term memory processes and cognitive functions were evaluated in the “Conditioned reflex of passive Avoidance” (CRPA) test (Table 3). During the first CRPA formation test, the animals of all groups entered the dark compartment of a chamber (100% reflex). On the second day of short-term memory testing, there were no significant differences between the groups. According to the latency of the entrance to the dark compartment, the animals of the IMM-FFI group were closer to the control group, i.e., they were characterized by fewer anxiety-like functions. However, this result was at the trend level. In general, the data obtained indicate the absence of either negative or positive effects from the developed FFI on the learning ability and memory of animals in the CRPA test.

#### 2.3.3. Biochemical Indices

Table 4 shows the results after determining the content of protein metabolism (total protein, albumin, globulins, urea, creatinine), lipid metabolism (total cholesterol, HDL cholesterol, LDL cholesterol, triglycerides), mineral metabolism (phosphorus), and liver function (total bilirubin, AlAT, AsAT) in the blood plasma of the rats. In animals of the IMM-FFI group treated with FFI, a significant decrease in the level of blood triglycerides was found compared to the CTRL and IMM groups (*p* < 0.05, Mann-Whitney test). The animals of the IMM group showed a significant increase in total bilirubin (*p* < 0.05, Mann-Whitney test). Consumption of the developed FFI completely leveled the growth of this indicator for animals in the experimental group. Daily immobilization of the IMM group reduced the pool of globulins in the blood of rats compared with intact animals of the control group; consumption of FFI positively affected this indicator, returning it to the values of the control group (*p* < 0.05, Mann-Whitney test). The remaining biochemical parameters of the blood plasma did not differ significantly between the groups.

**Figure 3:**
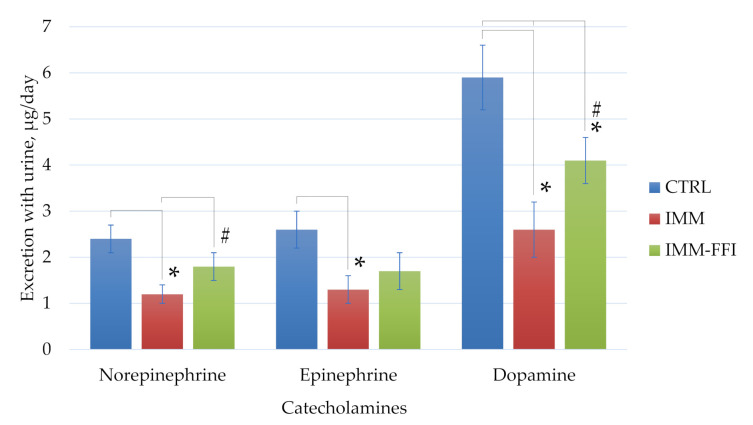
Daily urinary catecholamine excretion, µg/day. *—Differences are significant compared to the CTRL group, *p* < 0.05. **#**—Differences are significant compared to the IMM group, *p* < 0.05.

Figure 3 shows the results of catecholamine excretion in the daily urine of the animals. As can be seen from the data, immobilization led to a significant decrease in the content of dopamine, norepinephrine, and epinephrine in the daily urine of animals compared with the indicators for animals of the control group (*p* < 0.05, Mann-Whitney test). Consumption of FFI by rats positively affected the balance of the stress activators studied, and prevented depletion of the pool of biogenic amines in stressed animals. These differences were significant in the case of norepinephrine and dopamine levels (*p* < 0.05, Mann–Whitney test).

### 2.4. Experiment No. 2: Adaptogenic Properties of FFI in a Model of Increased Physical Energy Expenditure

#### 2.4.1. Integral Indicators

The results of the rats’ division into groups according to the EPM test and body weight of the animals are presented in Table 5.

The results of animal feed intake (Supplementary Figure S2) and body weight (Supplementary Figure S3) dynamics show no differences between the groups, in contrast to experiment No. 1, where daily immobilization was the cause of decreased dietary intake and lag in body weight. The FFI intake of the RUN-FFI experimental group animals was 91.1 ± 12.9 mg FFI per kg body weight per day, corresponding to 1.6 ± 0.1 mg 20E per kg body weight per day.

#### 2.4.2. Memory Function, Behavioral Responses, and Exhausting Physical Exercise on the Treadmill

Memory function and behavioral responses in the EPM and CRPA tests in rats in this experiment were evaluated to examine the effects of daily FFI consumption in the absence of daily model immobilization prior to the increased physical energy expenditure test. The 25-day consumption of FFI by rats did not affect anxiety-like functions (time in the open and closed arms of the EPM). In the EPM test, there were no differences between any of the groups in the indices of distance traveled and transitions between the arms of the maze (Supplementary Figure S4). During short-term memory testing in the CRPA test, there were no significant differences between the groups, which indicates the absence of FFI influence on the learning ability and memory of animals (Supplementary Table S1).

**Figure 4:**
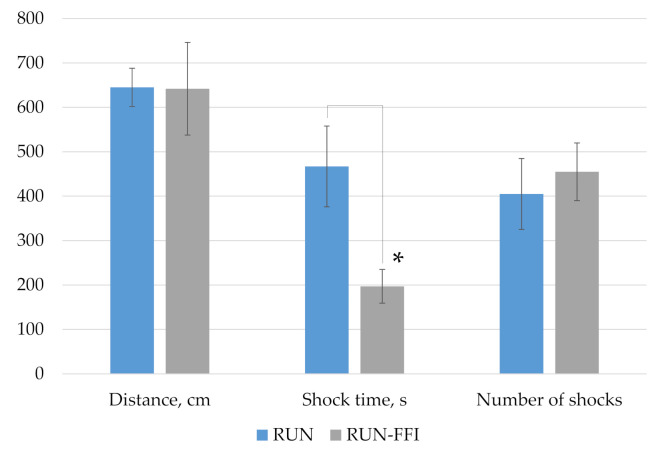
Results of testing rats on a treadmill. *—Differences are significant compared to the RUN group, *p* < 0.05.

Figure 4 shows the results obtained during the exhausting physical exercise test on a treadmill. At the end of testing, there were no significant differences in indicators such as distance traveled and number of electric shocks received between the compared groups. However, a significant increase in shock time, by more than two times, was found in animals of the RUN control group, compared with animals receiving FFI (*p* < 0.05, Mann–Whitney test). Thus, the consumption of FFI contributed to the improvement of physical performance and reaction rate in animals under simulated conditions.

#### 2.4.3. Biochemical Indices

Table 6 shows the results of the determination of biochemical parameters in the blood plasma of rats. The animals in the RUN group showed significant increases in total cholesterol level, AsAT activity, and phosphorus and protein content in the blood due to an increase in both fractions after a single exhausting physical activity. Consumption of FFI by rats over 25 days led to a radically different response to physical activity. In these animals, triglycerides were reduced by almost two times in comparison with the RUN group, and total cholesterol levels were increased, together with HDL and LDL fractions. Animals of the RUN-FFI group were characterized by increased activity of both transaminases, which indicates some intensification of the tricarboxylic acid cycle. However, when calculating the AsAT/ALAT index [32], this difference loses its significance due to the relatively small increase in the levels of both transaminases. It is noteworthy that in the RUN-FFI animals, protein metabolism indicators remained unchanged and phosphorus levels were below the level of the control group.

**Figure 5:**
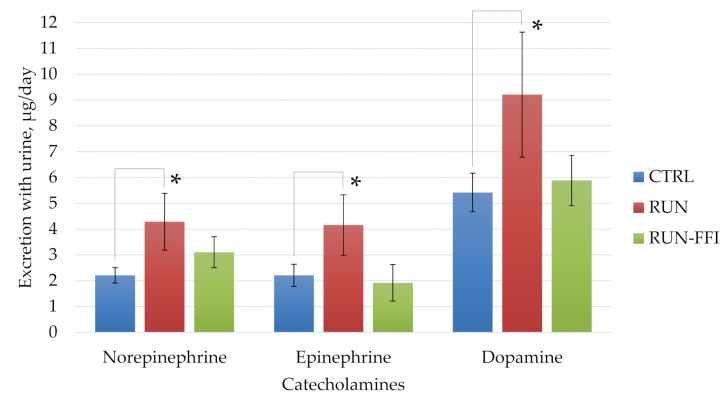
Daily urinary catecholamine excretion, µg/day. *—Differences are significant compared to the CTRL group, *p* < 0.05.

Figure 5 shows the results of daily urinary catecholamine excretion of animals. A significant increase in the content of all catecholamines in the urine of the RUN group animals compared to the RUN-FFI group animals was shown (*p* < 0.05, Mann–Whitney test). Consumption of FFI by the rats appears to have a positive effect on the balance of the stress activators studied, preventing an increase in their blood levels.

## 3. Discussion

Considering the impressive spectrum of biological activity of spinach, the synergistic aspect of the action of phytoecdysteroids and minor BAS of polyphenolic nature included in its composition is of particular interest. Quantification of 20E under controlled spinach cultivation conditions can range from 50 to 800 µg per g fresh tissue, depending on the growth rate [6]. The average rate of 20E is about 300 µg per g of fresh tissue [33]. However, such high concentrations of 20E do not characterize all varieties of spinach. In most species, the average amount of 20E ranges from 8.2 to 27 µg per g fresh tissue [34]. The authors of [24], using concentration and extraction techniques similar to ours [25], managed to achieve similar results in terms of total polyphenol content.

Despite the apparent safety of a plant such as spinach, high concentrations of some plant polyphenols may exhibit toxicological characteristics [35,36]. The available literature describes the few results of studies of LD_50_ glycoglycerolipid fractions of spinach extracts in cell cultures in vitro. In [37], no signs of toxicity were observed in ICR mice according to OECD protocol 420 [31] at doses from 300 to 2000 mg/kg of body weight. Investigation of acute oral toxicity of methanol and aqueous extracts of *Spinacia oleracea* L. in Swiss albino mice showed the absence of lethality and pathological signs in the dose range from 500 to 2000 mg/kg of body weight [38]. Taken together, it can be concluded that the profile of polyphenols contained in extracts of *Spinacia oleracea* L. have low toxicity and are classified as low-hazard substances.

An important role in the adaptation of animals to stress is played by individual behavioral differences, which have been confirmed by the results of previous studies [39,40]. In this study, we applied an experimental approach based on the preliminary differentiation of rats by behavioral phenotype according to their performance in the EPM test. The EPM test examines animal behavior under variable stress conditions, i.e., with a free choice of comfort conditions, which allows us to assess their level of anxiety-like functions. The behavioral assessment in the EPM is based on an animal’s natural tendency to stay in enclosed dark areas and natural fear of open spaces and heights. The EPM test allows estimation of the degree of expression of the emotional reactions of fear and anxiety as well as motor activity and the speed of orienting reactions [2].

The consumption of FFI by rats contributed to a pronounced anxiolytic effect in a model of immobilization-induced emotional stress. Daily immobilization reduced appetite in the rats of the IMM group, which led to a lag in their growth starting from the 10th day of the experiment. Consumption of FFI had a positive effect on animal feed intake and, maintained body weight despite these animals being characterized by a reduced level of triglycerides. The anxiolytic effect observed during EPM testing in animals that consumed FFI was manifested in a longer (three times compared to IMM) stay of rats in open arms, which indicates a decrease in their anxiety-like functions [41]. At the trend level, some anxiolytic effect was observed in the CRPA test, which manifested in a longer indicator of the entrance latency to the dark compartment of the chamber. A decrease in the latency index during the formation of a memory trace in the CRPA test characterized an increased anxiety-like function in these animals [42]. In the treadmill test, animals are forced to run by using an electrode placed at the lower end of the treadmill. The technique allows assessment of the degree of fatigue in rodents by registering the distance traveled, the number of electric shocks received, and the total time during which the electric shock occurred [43,44]. Consumption of FFI reduced the reaction time of animals by more than two times when touching the electrode, which against the background of the same distance traveled characterizes rats with greater adaptability to the treadmill. The results of the anxiolytic action are consistent with the work of [12], where the effect of water–alcohol spinach extract at doses of 200 and 400 mg/kg of body weight on the expression of TNF-α and IL-1β in the hippocampus of male Wistar rats exposed to chronic stress was studied. The results showed that the expression of IL-1β and TNF-α was increased in the hippocampus of rats exposed to stress. As a result of consuming the extract, the authors observed a decrease in neuroinflammation in the hippocampus. In [45], aging female rats of the Long–Evans line consumed dry spinach leaves at the rate of 8 g per kg of body weight. The animals demonstrated improved cognitive function and memory during spatial learning in the “Morris water maze” test. We can conclude that the 25-day consumption of FFI had a positive effect not only on anxiety-like functions but also on the reaction rate in the “Treadmill test”, as well as indicators of search and locomotor activity in the EPM test.

In both experiments, animals that consumed the FFI were characterized by significantly reduced blood triglycerides compared to the control and experimental RUN and IMM groups. Such results indicate a pronounced hypolipidemic effect of the developed FFI, regardless of the model. In some part, this is probably due to the increased activity of lipoprotein lipase, which increases under the influence of physical exertion, leading to a decrease in blood triglyceride levels [46]. However, this is most consistent with [13], where the antihyperlipidemic effects of spinach extract were evaluated using a model of aerobic exercise (swimming) in rats fed a high-fat diet. The authors concluded that spinach extracts inhibit pancreatic lipase in vivo in rats, preventing the intestinal absorption of food fat and thereby reducing weight gain-induced weight.

It should be noted that despite the hypolipidemic effect of FFI, the animals were characterized by normal body weight, and in the experiment with daily immobilization they even normalized it compared to the IMM-FFI group. This may indicate the presence of greater muscle mass in the rats that consumed the FFI for 25 days. All of this is consistent with the work of [34], who studied the effects of spinach on protein biosynthesis in skeletal muscles. A significant increase in protein synthesis in skeletal muscle cells was shown after treatment with spinach leaves. The authors linked the results of the anabolic activity with the presence of phytoecdysteroids in spinach. However, the authors also concluded that spinach contains other non-ecdysteroid compounds with anabolic activity. Based on this, there is an interesting dependence of the endurance of RUN-FFI rats on the treadmill to the quantitative levels of blood proteins, which remains normal under conditions of increased energy consumption. Only a few elevated levels of transaminases show more intense protein catabolism in these animals. Thus, it can be assumed that animals that consumed the FFI were physically more enduring due to the presence of greater muscle mass. This advantage allowed them to overcome the exhausting physical activity without much effort, which is also reflected in a slight increase in urinary excretion of catecholamines, the main activators of the stress system [47]. To clarify the features of the anabolic properties of FFI, it may be promising to conduct studies to assess the effects of a wide range of FFI dosages under physical exercise, with an assessment of changes in the dynamics of body weight composition by noninvasive nuclear magnetic resonance relaxometry methods together with assessment of specific markers.

## 4. Materials and Methods

### 4.1. Preparation and Characterization of the FFI from Spinacia oleracea L. Leaves

**Figure 6:**
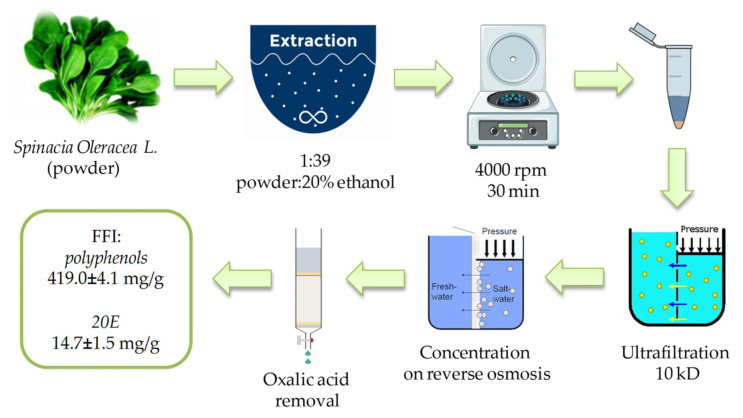
Flow chart of the FFI production from *Spinacia oleracea L*.

Fresh spinach (*Spinacia oleracea* L.) leaves were dried on a freeze-dryer LS 500 (“Prointech”, Pushchino, Russian Federation) and ground to powder using a laboratory blender (“FimarFRI”, Italy). The complex of phytoecdysteroids and polyphenols was extracted from the dry powder at 25 °C using an aqueous solution of 20% ethyl alcohol at a ratio of dry spinach powder/extractant of 1/39. After centrifugation for 30 min at 4000 rpm (BeckmanJ-6B centrifuge, (Indianapolis, IN, USA)) the supernatant was collected and filtered through a membrane with a pore diameter of 10 kD (a laboratory unit for micro- and ultrafiltration based on an ASF-018 filter holder, production “Vladisart”, Russian Federation). The collected low molecular weight fraction was concentrated in a reverse osmosis unit with a rolling membrane filter URF-1812 (manufactured by “Vladisart”, Russian Federation) with subsequent freeze-drying and removal of the oxalic acid by low-pressure chromatography. The flow chart of FFI production from *Spinacia oleracea* L. is presented in Figure 6.

The content of phytoecdysteroids (20E) was determined by HPLC-MS. Total polyphenols were determined spectrophotometrically by the Folin-Ciocalteu method. The total content and profile of individual flavonoids were determined by HPLC-DAD. Oxalic acid content at different stages of the FFI production was determined by permanganatometric titration. Detailed analysis procedures have been described previously [25].

### 4.2. Animals and Experimental Design

#### 4.2.1. Animals and Ethics

We used rats of the outbred Wistar line and mice of the outbred ICR (CD) line purchased from the Stolbovaya breeding nursery (Scientific Center for Biomedical Technologies, FMBA, Moscow region, Russian Federation). The animals were kept in pairs in polycarbonate cages under controlled environmental conditions (temperature 21–24 °C, relative humidity 30–60%, 12/12 h illumination conditions). Animals received a balanced semi-synthetic diet according to AIN93M [48] and drinking water purified by reverse osmosis (Merck-Millipore, Burlington, MA, USA). The work with animals adhered to the standard principles described in [49]. The studies were conducted according to the guidelines of the Declaration of Helsinki and approved by the Ethics Committee of the Federal Research Centre of Nutrition and Biotechnology (protocol code No. 02-19, 6 October 2019).

#### 4.2.2. Acute Oral Toxicity: Fixed Dose Procedure

The study of FFI acute toxicity was carried out according to the “420 OECD guideline for testing of chemicals” [31], with some modifications. Sixteen male and sixteen female mice (healthy young mature individuals at the age of five weeks) with an initial body weight of 26 ± 2 g and 24 ± 1 g, respectively, were used in the experiment. After one week of adaptation to vivarium conditions, mice were divided into four equal groups (*n* = 8): 1—control males, 2—experimental males, 3—control females, 4—experimental females. Experimental animals were administered FFI intragastrically through a probe at the rate of 5000 mg per 1 kg body weight. FFI was dissolved in drinking water purified by reverse osmosis. Control animals were given only water in the same volume. The animals were observed for six hours after FFI administration. General condition, motor activity, convulsions, tone of skeletal muscles, respiratory rate, depth of respiratory movements, condition of hair coat, condition of mucous membranes, stool, and urination were fixed. Further, the same parameters were assessed in mice daily for 13 days, and the dynamics of body weight weekly. Animals were removed from the experiment on the 14th day by CO_2_ inhalation, and the thoracic and abdominal cavity organs were examined. In addition, relative liver weight was assessed; the liver was sampled to study lipid metabolism parameters (cholesterol, triglycerides), and the blood in order to study corticosterone and prostaglandin E2 levels.

#### 4.2.3. Experiment No. 1: Study of FFI Adaptogenic Properties in a Model of Immobilization-induced Emotional Stress

The experiment was carried out using 40 growing male Wistar rats at the age of four weeks with an initial body weight of 80 ± 5 g. Before the experiment, a behavioral phenotype should be determined in rats to differentiate them on the basis of active or passive behavior. Rats with different behaviors demonstrate different adaptive responses to stress. Preliminary separation of animals into groups similar in behavior increases the verifiability of the obtained results [39,40]. For this purpose, after a seven-day quarantine, rats were separated depending on their behavioral phenotype in the “Elevated Plus Maze” (EPM) test. The duration of time in the maze was five minutes. During testing, the number of arms visited, the time spent in closed arms (CA) and open arms (OA), and the locomotor activity were recorded. To assess the dynamics of changes in the degree of anxiety-like functions, repeated testing in the EPM was carried out on the 25th day of the experiment. Rats’ activity in the maze was recorded using the Smart 3.0.04 video surveillance system (Panlab Harvard Apparatus, Spain). Characteristics of the EPM used and the procedure for assessing the level of anxiety-like behavior were described earlier in [41].

Animals were randomly divided by body weight and EPM test results into three groups: CTRL (*n* = 20), IMM (*n* = 10), and IMM-FFI (*n* = 10). Animals of the CTRL and IMM groups received a standard semi-synthetic diet for 26 days of the experiment. FFI was added to the diet of IMM-FFI group animals in the amount of 600 mg per 100 g. In all groups, the diets were iso-caloric and iso-nitrogenic. The animals received food and water ad libitum. Feed intake was assessed every other day. The body weight of the animals was recorded on days 5, 7, 12, 18, and 25.

Animals of the IMM and IMM-FFI groups were subjected to daily immobilization throughout the experiment (26 days) by placing them in transparent houses-fixators that restrict freedom of movement (AE1001-R1, Open Science LLC, Russian Federation). The duration of immobilization was 40 min.

The assessment of the behavior and short-term memory of animals was carried out on the 15th day of the experiment using the “Conditioned reflex of passive Avoidance” (CRPA) test according to the methodology and on equipment described earlier [41].

**Figure 7:**
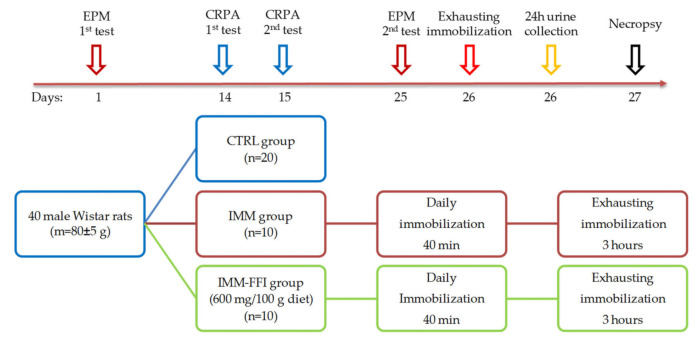
Experiment No.1. design and timeline.

On the 26th day of the experiment, the animals of the IMM and IMM-FFI groups were subjected to exhausting immobilization for 3 h. Immediately after exhausting immobilization, the animals were placed for 24 h in metabolic cages to collect daily urine. On day 27, fasted rats were removed from the experiment by decapitation under light ether anesthesia. The blood of the animals was collected on an anticoagulant and centrifuged for 15 min at 4000 rpm. The rats’ plasma was stored at −80 °C. Catecholamine content in the urine was analyzed, as were the blood-indices of protein, lipid, and mineral metabolism and indices of the functional state of the liver. The design scheme and timeline are shown in Figure 7.

#### 4.2.4. Experiment No. 2: Study of FFI Adaptogenic Properties in a Model of Increased Physical Energy Expenditure

**Figure 8:**
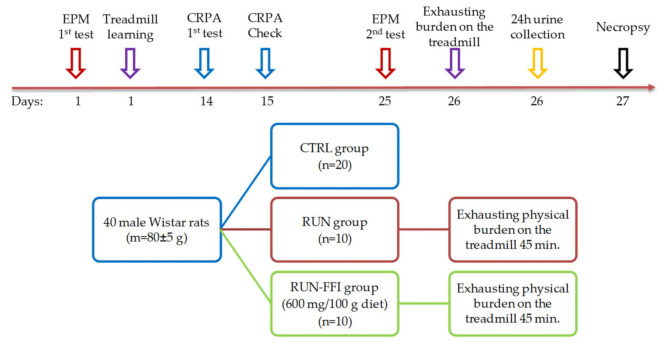
Experiment No.2 design and timeline.

Sixty male Wistar rats (at the age of four weeks) with an initial body weight of 80 ± 5 g were taken before the experiment. As in experiment No. 1, before the study animals were divided by their behavioral phenotype in the EPM test after quarantine. According to [43], when the treadmill test (Treadmill, PanLab, Barcelona, Spain) is used in the model, it is recommended to train experimental animals at low speeds, inclinations, and durations to avoid unintentional injury. The duration of the training was 10 min; belt speed was gradually increased from 15 cm/s to 18 cm/s with a treadmill incline of 0°. Further, according to [44], the learning ability of the animals was evaluated. Rats not capable of running were excluded from the experiment. Afterwards, 40 animals were randomly divided into three groups according to their body weight, EPM test results, and treadmill training: CTRL (*n* = 20), RUN (*n* = 10), and RUN-FFI (*n* = 10). Animals of groups CTRL and RUN received a standard semi-synthetic diet for 26 days of the experiment. Rats of the RUN-FFI group received a modified semi-synthetic diet supplemented with FFI in amount 600 mg per 100 g. The animals received food and water ad libitum. Feed intake was assessed every other day. The body weight of the animals was recorded on days 5, 7, 12, 18, and 25. Assessment of the behavior and short-term memory of animals was carried out on the 15th day of the experiment using the CRPA test. On the 26th day of the experiment, the CTRL, RUN and RUN-FFI animals were all subjected to the exhausting physical burden test on the treadmill; over a 45 min duration, belt speed was gradually increased from 26 cm/s to 40 cm/s, and treadmill incline was 10°. Immediately after running, the animals were placed in metabolic cages for 24 h to collect daily urine in order to study catecholamine excretion levels. On day 27, fasted rats were removed from the experiment by decapitation under mild ether anesthesia; blood was collected to study biochemical parameters. The design scheme and timeline are shown in Figure 8.

### 4.3. Assessment of Biochemical Indices

The protein metabolism (total protein, albumin, globulins), lipid metabolism (total cholesterol, HDL cholesterol, LDL cholesterol, triglycerides), mineral metabolism (phosphorus), and liver functional state (total bilirubin, AlAT, AsAT, AsAT/AlAT) were determined in blood plasma using an automatic biochemical analyzer “Konelab 20i” (Thermo Fisher Scientific, Waltham, MA, USA) according to standard methods [42].

The content of norepinephrine, epinephrine, and dopamine (catecholamines) in urine was determined by HPLC. Sample preparation was carried out as follows: urine (5 mL) was centrifuged (4000 rpm, 30 min), then 1.0 M Tris-HCl buffer (pH 8.6) brought the pH of the sample to 8.5 according to the universal pH-meter, and 40 mL of an internal standard solution, 3,4-dihydrobenzylamine hydrobromide (“Sigma-Aldrich”, Burlington, MA, USA) was added and quantitatively applied to a micro-column (0.5 × 1.0 cm) with aluminum oxide. The sorbent was washed with distilled water (2 × 2 mL); catecholamines and 3,4-dihydrobenzylamine hydrobromide were eluted with a 1.0 M acetic acid solution. The resulting eluent was injected into a chromatographic column (Nucleodur C18, 5 microns, 250 × 5 mm), pre-calibrated according to an internal standard (“Sigma-Aldrich”, Burlington, MA, USA). The composition of the mobile phase was 0.1 M phosphate-citrate buffer pH 4.0, containing 50 mg/l of ion-paired reagent (1-octanesulfonic acid sodium salt for HPLC, Dudley Chemical, Lakewood, NJ, USA) and 2.5% acetonitrile (qualification “for HPLC”). The volume of the injected sample was 100 µL. The elution rate was 1.0 mL/min. An amperometric detector was used (Khimavtomatika, Moscow, Russian Federation) with a glass-carbon electrode and an operating voltage of +1.0 V.

In the acute oral toxicity experiment, blood prostaglandin E2 levels were determined by quantitative competitive immunoassay using a commercial reagent kit according to the manufacturer’s methodology (R&D systems, Minneapolis, MN, USA). Corticosterone content was determined in blood plasma by quantitative competitive immunoassay using a commercial reagent kit according to the manufacturer’s methodology (IDS Limited, West Boldon, UK).

### 4.4. Statistical Analysis

Statistical processing was carried out using the SPSS 24.0 package and Microsoft Excel for Windows. The calculation included the determination of the sample mean (M), standard error (s.e.m.), and standard deviation (SD). Data are presented as M ± s.e.m. The probability of accepting the null hypothesis about the coincidence of the distributions of the compared samples was established according to the two-sided Student’s t-test for pairwise related values, post hoc Wilcoxon Mann–Whitney nonparametric tests, Kruskal–Wallis, and ANOVA criteria. The differences were recognized as significant at a significance level of *p* < 0.05.

## 5. Conclusions

The FFI developed for this study is a rich source of polyphenolic BAS and a highly concentrated extract containing 30 times more 20E than the fresh leaves of *Spinacia oleracea* L. In the conditions of the reproduced models, FFI demonstrated an anxiolytic effect, positively influenced lipogenesis, normalized the excretion of catecholamines in urine, and had a pronounced anabolic effect. Thus, the studied safety, adaptogenic and anabolic properties of the FFI make it a promising component of specialized food products for conducting clinical trials of products in certain categories of humans subjected to psychoemotional stress and increased physical activity. Due to the lack of oxalates in FFI, such products will be able to find their use in the future, including in people suffering from or prone to kidney stones, rheumatism and gout.

## Figures and Tables

**Table 1:** Flavonoid content in the developed FFI.

Flavonoid	Content, %
The sum of flavonoids	44.0 ± 2.0
Patuletin-3-glucosyl-(1→6)-apiosyl-(1→2)-glucoside	5.5 ± 0.8
Patuletin-3-glucosyl-(1→6)-glucoside	0.9 ± 0.3
Patuletin-3-(2″feruloylglucosyl)-(1→6)-apiosyl-(1→2)-glucoside	3.1 ± 0.4
Patuletin-3-(2″feruloylglucosyl)-(1→6)-glucoside	1.3 ± 0.3
Axilyarin-4′-glucuronide (spinatoside)	3.8 ± 0.6
5,3′,4′-trihydroxy-3-methoxy-6:7-methylenedioxyflavone-4′-glucuronide	23.4 ± 0.4
5,4′-dihydroxy-3-methoxy-6:7-methylenedioxyflavone-4′-β-D-glucuronide	1.5 ± 0.2
5,4′-dihydroxy-3,3′-dimethoxy-6:7-methylenedioxyflavone-4′-glucuronide	1.0 ± 0.2

Note: Here and further in the text, the values are presented as means ± standard error.

**Table 2:** Group formation according to the EPM test and body weight of rats.

Indicator	Group		
	CTRL	IMM	IMM-FFI
Body weight, g	104 ± 3	103 ± 3	105 ± 2
Time in open arms, s	61 ± 9	58 ± 10	63 ± 11
Time in closed arms, s	206 ± 12	206 ± 13	204 ± 15
Distance, cm	1533 ± 112	1553 ± 100	1556 ± 168
Number of transitions	26 ± 3	26 ± 1	30 ± 4

**Table 3:** Behavioral reactions of rats in the CRPA test.

	First Test CRPA Formation		Second Test after 24 h Short-Term Memory	
	Latency, s	Non-Entered Animals (Excluded from the Test)	Latency, s	Number of Animals Entered
CTRL	40 ± 10	2 (20%)	122 ± 18	3 (30%)
IMM	34 ± 8	0 (0%)	105 ± 25	4 (40%)
IMM-FFI	48 ± 12	1 (10%)	145 ± 23	2 (20%)

**Table 4:** Biochemical blood indicators of rats.

Indicator	Group		
	CTRL	IMM	IMM-FFI
HDL, mmol/l	0.58 ± 0.03	0.66 ± 0.08	0.60 ± 0.05
LDL mmol/l	0.17 ± 0.01	0.15 ± 0.01	0.15 ± 0.02
Triglycerides, mmol/l	0.68 ± 0.05	0.84 ± 0.09	0.46 ± 0.05 ^1,2^
Cholesterol, mmol/l	1.24 ± 0.02	1.31 ± 0.07	1.24 ± 0.09
AlAT, U/l	45.5 ± 1.6	42.5 ± 2.5	48.7 ± 3.4
AsAT, U/l	119.6 ± 4.7	116.5 ± 9.0	132 ± 11.6
Total bilirubin, mmol/l	2.96 ± 0.14	3.57 ± 0.30 ^1^	2.65 ± 0.13 ^2^
Globulin, g/l	19.3 ± 0.4	17.9 ± 0.4 ^1^	19.3 ± 0.8
Albumin, g/l	24.4 ± 0.2	24.8 ± 0.3	24.1 ± 0.7
Total protein, g/l	43.6 ± 0.5	42.5 ± 0.6	43.4 ± 1.5
Phosphorus, mmol/l	1.95 ± 0.04	1.92 ± 0.06	1.89 ± 0.07

Note: ^1^—differences are significant against CTRL group; ^2^—differences are significant against IMM group, *p* < 0.05. HDL—high-density lipoproteins; LDL—low-density lipoproteins; AlAT—alanine aminotransferase; AsAT—aspartate aminotransferase.

**Table 5:** Groups’ formation according to the EPM test and body weight of rats.

Indicator	Group		
	CTRL	RUN	RUN-FFI
Body weight, g	104 ± 3	103 ± 3	104 ± 3
Time in open arms, s	61 ± 9	58 ± 10	62 ± 7
Time in closed arms, s	206 ± 12	206 ± 13	206 ± 11
Distance, cm	1533 ± 112	1553 ± 100	1553 ± 157
Number of transitions	26 ± 3	25 ± 3	27 ± 3

**Table 6:** Biochemical blood indicators of rats in experiment No. 2.

Indicator	Group		
	CTRL	RUN	RUN-FFI
HDL, mmol/l	0.58 ± 0.03	0.65 ± 0.05	0.69 ± 0.03 ^1^
LDL mmol/l	0.17 ± 0.01	0.18 ± 0.01	0.25 ± 0.03 ^1^
Triglycerides, mmol/l	0.68 ± 0.05	0.83 ± 0.08	0.45 ± 0.03 ^1,2^
Cholesterol, mmol/l	1.24 ± 0.02	1.50 ± 0.07 ^1^	1.45 ± 0.03 ^1^
AlAT, U/l	45.6 ± 1.6	47.0 ± 2.4	57.3 ± 3.3 ^1,2^
AsAT, U/l	119.7 ± 4.7	140.0 ± 8.2 ^1^	147.4 ± 12.1 ^1^
AsAT/AlAT	2.67 ± 0.12	3.03 ± 0.66	2.56 ± 0.29
Total bilirubin, mmol/l	2.96 ± 0.14	2.81 ± 0.19	2.59 ± 0.21
Globulin, g/l	19.3 ± 0.4	21.5 ± 0.7 ^1^	20.2 ± 0.3 ^1,2^
Albumin, g/l	24.3 ± 0.2	25.1 ± 0.3 ^1^	24.6 ± 0.4
Total protein, g/l	43.6 ± 0.5	46.7 ± 0.9 ^1^	44.8 ± 0.6
Phosphorus, mmol/l	1.95 ± 0.04	2.08 ± 0.04 ^1^	1.88 ± 0.06 ^2^

Note: ^1^—differences are significant against CTRL group; ^2^—differences are significant against IMM group, *p* < 0.05. HDL—high-density lipoproteins; LDL—low-density lipoproteins; AlAT—alanine aminotransferase; AsAT—aspartate aminotransferase.

## Data Availability

Data available on request due to restrictions e.g., privacy or ethical.

## Supplementary Materials

The following are available online at https://www.mdpi.com/article/10.3390/plants10122555/s1. Figure S1: Acute toxicity experiment indicators; Figure S2: Rats body weight during experiment No. 2; Figure S3: Food consumption by rats in experiment No. 2; Figure S4: Results of the EPM testing of rats in experiment No. 2; Table S1: Rats’ short-term memory testing in the CRPA test.
